# Differences in postural strategies between children with and without ADHD in tasks of static and dynamic balance

**DOI:** 10.3389/fnhum.2025.1630049

**Published:** 2025-08-01

**Authors:** Tabea Christ, Kim Joris Boström, Heiko Wagner, Christiane Bohn

**Affiliations:** Department of Movement Science, University of Münster, Münster, Germany

**Keywords:** ADHD, children, balance tasks, postural control, joint torques, postural strategies

## Abstract

**Background:**

Many children with ADHD experience challenges with balance and postural control, unlike their unaffected peers. While postural sway has been extensively studied in this patient group, less is known about the postural strategies employed to maintain equilibrium. This study extends the examination of hip and ankle postural strategies by including an upper body strategy involving movements of the head, arms, and trunk to regain balance. The aim was to investigate the differences in postural control strategies between children with and without ADHD.

**Methods:**

Forty one children (17 diagnosed with ADHD, 24 unaffected controls) with a mean age of 10.0 ± 1.4 years participated in the study. For the assessment of static balance, a 25-s one-leg stand was conducted. Dynamic balance was evaluated by balancing on a narrow wooden beam. Kinetic data was recorded using Kistler force plates. Kinematic data was collected with the Qualisys motion capture system. Joint torque amplitudes were calculated using an inverse-dynamics approach employing the Myonardo software.

**Results:**

Children with ADHD produced significantly higher joint torques during static and dynamic balancing compared to neurotypical children in the ankle and upper body joint group. Torque amplitudes of the upper body joints were 11% and 7.5% higher for the dynamic and static balance tasks, respectively, and 22% and 20% higher for the ankle joint. For hip joint torque variation, the ADHD group exhibited 25% and 34% lower joint torques for the dynamic and static balance tasks, respectively.

**Conclusion:**

Impaired proprioception and neuromuscular control are common in children with ADHD and may present as reduced precision in muscle activation. Cerebellar deficits in this patient group negatively affect balance and coordination. Such deficits likely interfere with the adjustment of joint torques involved in maintaining balance and stability. Children with ADHD appear to struggle to efficiently employ and combine postural strategies as required by the given task. Assessments of balance and postural control in children with ADHD are indispensable for developing tailored interventions and thus reducing injury risk and promote wellbeing.

## 1 Introduction

Attention deficit hyperactivity disorder (ADHD) belongs to the most common neurodevelopmental childhood disorders worldwide (Tamm, 2009). Affected individuals typically present symptoms such as hyperactivity, impulsiveness, and inattention oftentimes leading to reduced quality of social functioning (Tamm, 2009). Additionally, studies have shown that many children with ADHD perform inferiorly in tests of fine and gross motor skills compared to age-matched neurotypical controls (Pitcher et al., 2003; Tseng et al., 2004).

Motoric ability, especially postural control and balance in ADHD patients have previously been investigated (Raberger and Wimmer, 2003; Ghanizadeh, 2011; Mao et al., 2014; Goetz et al., 2017). Difficulties in static and dynamic balance tasks among affected children likely stem from executive control problems and suggest a common cerebellar dysfunction (Raberger and Wimmer, 2003; Goetz et al., 2017). Interestingly, Schlee et al. (2012) did not find any deficiencies in ADHD patients compared to neurotypical children measured in a single and double-legged static balance test, which may, however be owed to the easiness of the tasks.

Balance ability is essential for many aspects of daily life, like riding a bike or engaging in physical activity. Balance, or more so postural control, can be defined as the act of maintaining, achieving, or restoring a state of balance during any posture or activity, and therefore constitutes a fundamental motor function (Pollock et al., 2000). Integrating inputs from the somatosensory, visual, and vestibular systems and using these sensory signals to generate coordinated motor actions and maintain body equilibrium is a process that may be impaired in ADHD patients (Kandel et al., 2000).

While balance ability of ADHD-affected children has been extensively studied in the past (Pitcher et al., 2003; Wang et al., 2003; Shum and Pang, 2009; Konicarova et al., 2014; Mao et al., 2014; Christ et al., 2024), little is known about postural strategies used by the affected group to maintain balance. The hip and ankle postural strategies have previously been identified and explored in the literature among a healthy population (Nashner and McCollum, 1985; Runge et al., 1999; Guihard and Gorce, 2002; Boonstra et al., 2013). While the ankle strategy is commonly described as a single-segment inverted pendulum and is predominantly used during activities that require little balance regulation, the hip strategy is characterized as a double-segment inverted pendulum and is observed during more complex balance tasks involving unstable and narrow surfaces or backward translation (Horak and Nashner, 1986; Runge et al., 1999).

In addition, a so-called upper body strategy, with regards to movements of the head, arms, and trunk, seems to play an essential role in maintaining balance (Hsu et al., 2007; Pinter et al., 2008; Kilby et al., 2015; Boström et al., 2018). This is not surprising as moving or stretching out the arms to remain or restore equilibrium is a much-employed tactic in challenging balance tasks, which is supported by the literature (Milosevic et al., 2011; Shafeie et al., 2012; Cheng et al., 2014; Patel et al., 2014; Boström et al., 2018; Objero et al., 2019). Arm raises have been associated with an elevation of the center of mass (COM) position (Roos et al., 2008) as well as with a forward shift of the COM after its displacement due to a fall (Marigold et al., 2003) suggesting that movements of the upper body play a vital role for example in the design of fall prevention programs for the elderly (Allum et al., 2002; Marigold et al., 2003; Roos et al., 2008; Objero et al., 2019).

Postural strategies are typically defined by joint torques, ground reaction forces (GRF), and electromyography (EMG) activations (Runge et al., 1999; Clifford and Holder-Powell, 2010). Especially joint torques seem to be a valuable measure, as they can indicate how body movements are generated (Runge et al., 1999). While most studies assessing postural control and balance ability in children with ADHD draw conclusions based on postural sway (Wang et al., 2003; Shum and Pang, 2009; Shorer et al., 2012; Amini et al., 2018), a measure based on body kinematics, this study analyzes a less frequently used kinetic measure based on body forces, namely joint torque.

Studying the use of hip and ankle postural strategies, supported by an upper-body strategy, as postulated by Boström et al. (2018), may not only provide important awareness and knowledge for fall risk reduction in older adults, but may also contribute to the understanding of balance strategies used by children with and without ADHD. Recent meta-analysis underline that children with ADHD have an increased risk of injury, which is likely owed to deficits in motor control (Amiri et al., 2017; Ruiz-Goikoetxea et al., 2018). The aim of this study was to investigate the differences in postural strategies used by children with ADHD and typically developing children of the same age during static and dynamic balance tasks. To the best of our knowledge, this is the first attempt to do so, and thus the findings may be of great practical relevance for injury prevention and wellbeing within the affected group. To quantify differences in balance strategies between the two groups, the following two-tailed hypotheses were tested.

(1) There is a significant difference in torque amplitude and variation between children with ADHD and neurotypical children in all three joint groups tested during a static balance task.
(2) There is a significant difference in torque amplitude and variation between children with ADHD and neurotypical children in all three joint groups tested during a dynamic balance task.

## 2 Methods

### 2.1 Participants

Forty-one children aged between eight and 12 years (10.0 ± 1.4 years) were recruited via newspaper ads, social media advertisements, announcements in schools, the district government of Münster, and publications via the Skate Aid homepage. Seventeen children were diagnosed with ADHD by a pediatrician (15 boys, 2 girls, age: 10.6 ± 1.5 years, body height: 148 ± 10 cm, body mass: 38.8 ± 8.5 kg) representing the ADHD group. Twenty-four neurotypical children (14 boys, 10 girls, age: 9.6 ± 1.1 years, body height: 142 ± 9 cm, body mass: 33.9 ± 7.8 kg) constituted the control group. Children with a regular intake of medication with methylphenidate and those above the age of 13 participated in the larger framework of the research project. However, they were excluded from the statistical analysis in the present study to ensure that postural stability is not influenced by symptom-alleviating medication. The study was approved by the University of Münster ethics committee of faculty 7 (2018-10-HW), and written informed consent was obtained from the legal guardian of every participant.

### 2.2 Experimental procedure

The test comprised measurements of static and dynamic balance ability, performed barefoot. For the static balance task, participants were asked to stand as still as possible on one leg for 25 s. To familiarize with the task, a measurement of 30 s was timed, whereby the first 5 s were excluded from the analysis. The test was performed once for each leg on a stationary force plate (Kistler Group, Sindelfingen, Germany), 90 x 60 centimeters in size, recording GRF and center of pressure (COP) positions at 1,000 Hz. For the following dynamic balance task, participants were instructed to balance across a five-centimeter-wide, five-centimeter-high wooden balance beam. The beam was fixed to the floor with tape and had a total length of eight meters. Four subsequently arranged force plates measured kinetic data between meters three and six of the balance beam. Each subject performed this task six times and was randomly assigned to start the trial with either the right or left foot. No specific arm posture or balancing speed was required from the participants. One practice trial was granted before the actual measurement began. In case a participant lost balance and slipped off the beam, they were instructed to continue the test from the position they fell off. Additionally, full-body kinematic data was gathered for both tasks using the Qualisys motion capture system and Qualisys Track Manager software (Oqus 500, Qualisys AB, Gothenburg, Sweden, 2018–2022 versions). The setup comprised 18 high-speed infrared cameras tracking passive optoelectronic reflective markers at a sampling rate of 200 Hz. A set of 59 reflective markers, 20 mm in diameter, were placed on anatomical landmarks following a modified Helen Hayes marker set. The force plates and the kinematic system were synchronized with an external trigger signal (Qualisys AB, Gothenburg, Sweden).

### 2.3 Data processing

#### 2.3.1 Biomechanical model

To calculate torque amplitudes and variations, the recorded kinematic data were imported into the musculoskeletal model Myonardo^®^ (version 7.0.0, Predimo GmbH, Münster, Germany; Wagner et al., 2023), a whole-body model consisting of 23 segments, 682 muscle-tendon units, and 23 joints with 66 degrees of freedom. Myonardo^®^ is programmed in MATLAB 2023a (The MathWorks, Inc., Natick, Massachusetts, United States) using the Simscape Multibody 3D simulation environment. The mass and inertia of each segment were scaled to the height and mass of the subject (Hatze, 1980; Winter, 2009). For the current analysis, Myonardo^®^ was used to calculate GRF based on the acceleration of the COM and the mass (Wagner et al., 2024). For both balance conditions, an inverse dynamics simulation was done to calculate joint torques, by creating the following joint groups, in which individual joints were averaged. Ankle joint group: left ankle joint, right ankle joint. Hip joint group left hip joint, right hip joint. Upper body joint group: left clavicle joint, right clavicle joint, left shoulder joint, right shoulder joint, left elbow joint, right elbow joint, left wrist joint, right wrist joint, lumbar joint, neck joint.

To account for the inertial characteristics of children, the centers of gravity and radii of gyration of the children's body parts were taken from the literature and applied to the model, individually scaled to the anatomy of each participant (Li and Dangerfield, 1993).

#### 2.3.2 Balance performance measures

Following Boström et al. (2018), we used torque amplitude and variation as two separate measures of balance performance, representing different aspects of postural control activity. While the torque amplitude of a given joint more directly quantifies the amount of torque applied to the joint, on average, within the observed time interval of task completion, the torque variation quantifies the amount of change in torque, on average, within the observed time interval of task completion. Mathematically, the torque amplitude is equal to the time average of the absolute torque applied to the joint during each measurement, while the torque variation is equal to the standard deviation instead of the average. For the upper body joint group, the values for the torque amplitude of the individual joints in the group were averaged, similarly for the torque variation.

### 2.4 Statistical analysis

Statistical analyses were performed in MATLAB (version 2023a, The MathWorks, Inc., Natick, Massachusetts, USA). We used generalized linear mixed models (GLMM) instead of traditional ANOVA for three reasons. First, GLMM handles uneven group sizes caused by dropouts without discarding data or averaging, preserving statistical power. Second, it accommodates the mix of within- and between-subject variables, categorical variables, and continuous covariates by incorporating random effects, including random intercepts and slopes, for better model fit. Third, GLMMs are robust to non-normal data. A separate model fit was performed for each of the two tasks, i.e., the static and the dynamic balance task, and for each of the two dependent variables, i.e., torque amplitude and torque variation. A gamma distribution with a log link function and maximum pseudo-likelihood model fitting was used for all model fits. The Wilkinson formula of each model fit was given by


$$\begin{array}{r} \text{Y}\sim \text{Age}+{\text{ADHD}}^{*}\text{JointGroup}+(\text{ADHD}+\text{JointGroup}\\ +\text{Age}|\text{Subject}), \end{array}$$

where “Y” is the dependent variable representing either torque amplitude or torque variation, “Subject” is a random variable indicating the identity of the subject, “ADHD” indicates whether the subject was in the ADHD group, “JointGroup” has the levels “ankle,” “hip” and “upperBody,” and “Age” is a continuous covariate representing the subject's age. Since outliers can substantially distort the model fit, any point outside 1.5 times the interquartile range (0.25, 0.75), most likely due to measurement artifacts, was considered an outlier and removed before entering the model fit. That way, 5.3% and 5.7% of the data were removed for the static and dynamic balancing tasks, respectively. All other data, including those of faulty trials, were included into the analysis to not bias against the nature of the disorder and its symptoms. To aid in the interpretation of the results of the *post-hoc* comparisons, we calculated the percentage change between the pre- and post-tests for each pair, denoted by “%diff”.

After each model fit, we performed statistically corrected, multiple pairwise comparisons (*post-hoc* tests) based on the estimated marginal means (EMMs) using the *emmeans* package for Matlab (version 1.0.0 by John Hartman, available at https://github.com/jackatta/estimated-marginal-means). These EMMs were calculated from the GLMM, hence the data plots show these EMMs in the center of the violin plots rather than the mean or median. The level of statistical significance was set at α = 0.05. Effect sizes are interpreted after Cohen's rule of thumb, with the usual limits for partial eta squared 0.01 = small, 0.06 = medium, 0.14 = large (Cohen, 1973).

## 3 Results

ADHD-affected children generated *joint torque amplitudes* different from those of their typically developing peers. This is shown by the statistically significant main effect *ADHD* for both, the one-leg stand (*F* = 5.49, *p* = 0.02, $\eta_{p}^{2}$ = 0.02) and the balance beam task (*F* = 6.39, *p* = 0.01, $\eta_{p}^{2}$ = 0.009). The analysis yielded no significant differences between children with and without ADHD in terms of *joint torque variance* for both tasks. Strongly significant main effects for the *JointGroup* variable across all conditions were expected and indicate the dissimilarity in torque generation for the joint groups analyzed. Furthermore, the covariate *Age* had a significant effect on the torque amplitude measures, but not on the torque variation for each task (Table 1).

**Table 1:** Results of the linear model fit comparing children with and without ADHD for torque amplitude and variation for each task.

	**Term**	***F***	***p***	*** $\eta_{p}^{2}$ ***	**Effect size**
**One-leg stand torque amplitude**	ADHD	5.49	**0.02** ^*^	0.02	Small to medium
	Joint Group	1,474.87	**0** ^***^	0.93	Very large
	Cov. Age	4.65	**0.03** ^*^	0.02	Small
	ADHD × Joint Group	10.01	**< 0.001** ^***^	0.08	Medium to large
**One-leg stand torque variation**	ADHD	0.65	0.42	0.003	Very small
	Joint Group	453.4	**0** ^***^	0.8	Very large
	Cov. Age	0.41	0.52	0.002	Very small
	ADHD × Joint Group	3.79	**0.02** ^*^	0.03	Small to medium
**Balance beam torque amplitude**	ADHD	6.39	**0.01** ^*^	0.009	Small
	Joint Group	1,732.42	**0** ^***^	0.83	Very large
	Cov. Age	6.13	**0.01** ^*^	0.009	Small
	ADHD × Joint Group	0.99	0.37	0.003	Very small
**Balance beam torque variation**	ADHD	0.1	0.76	0.0001	Very small
	Joint Group	1,351.76	**0** ^***^	0.8	Very large
	Cov. Age	0.11	0.74	0.0002	Very small
	ADHD × Joint Group	5.39	**0.005** ^**^	0.02	Small

Significant results are in bold font and gray shaded, effect sizes are interpreted according to Cohen's rule of thumb and indicate how much of the effect can be explained by the given term. Asterisks denote the corresponding level of significance: ^*^p < 0.05, ^**^p < 0.01, ^***^p < 0.001.

**Figure 1:**
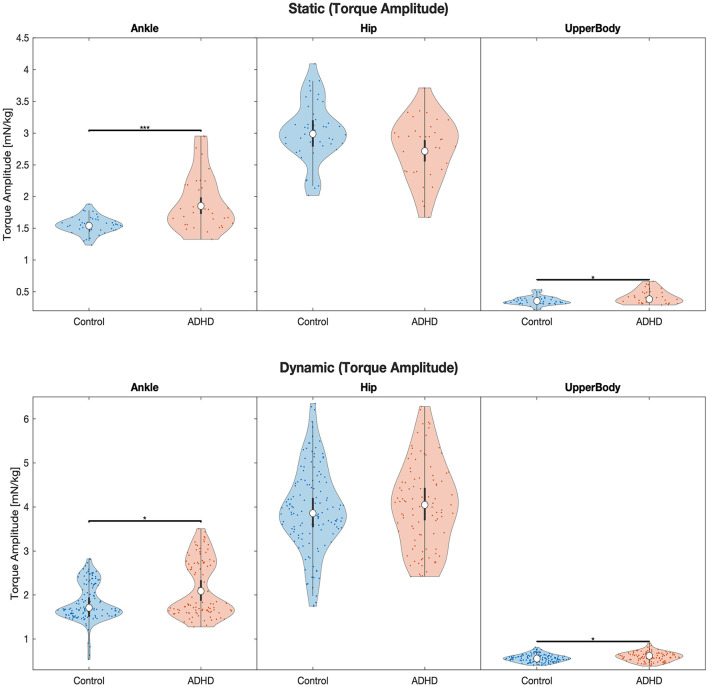
Violin plots of the linear model fit of joint torque amplitude for each joint group during static and dynamic balance tasks comparing children with and without ADHD. Empty circles indicate the estimated marginal mean (EMM), error bars denote the corresponding level of significance: ^*^p < 0.05, ^**^p < 0.01, ^***^p < 0.001. The units are in torque multiplied by 1,000 to obtain a more convenient number scale, giving Millinewton (mN) per meter body height and kilogram body mass, i.e., 1 mN^*^m/(kg^*^m) = 1 mN/kg.

**Figure 2:**
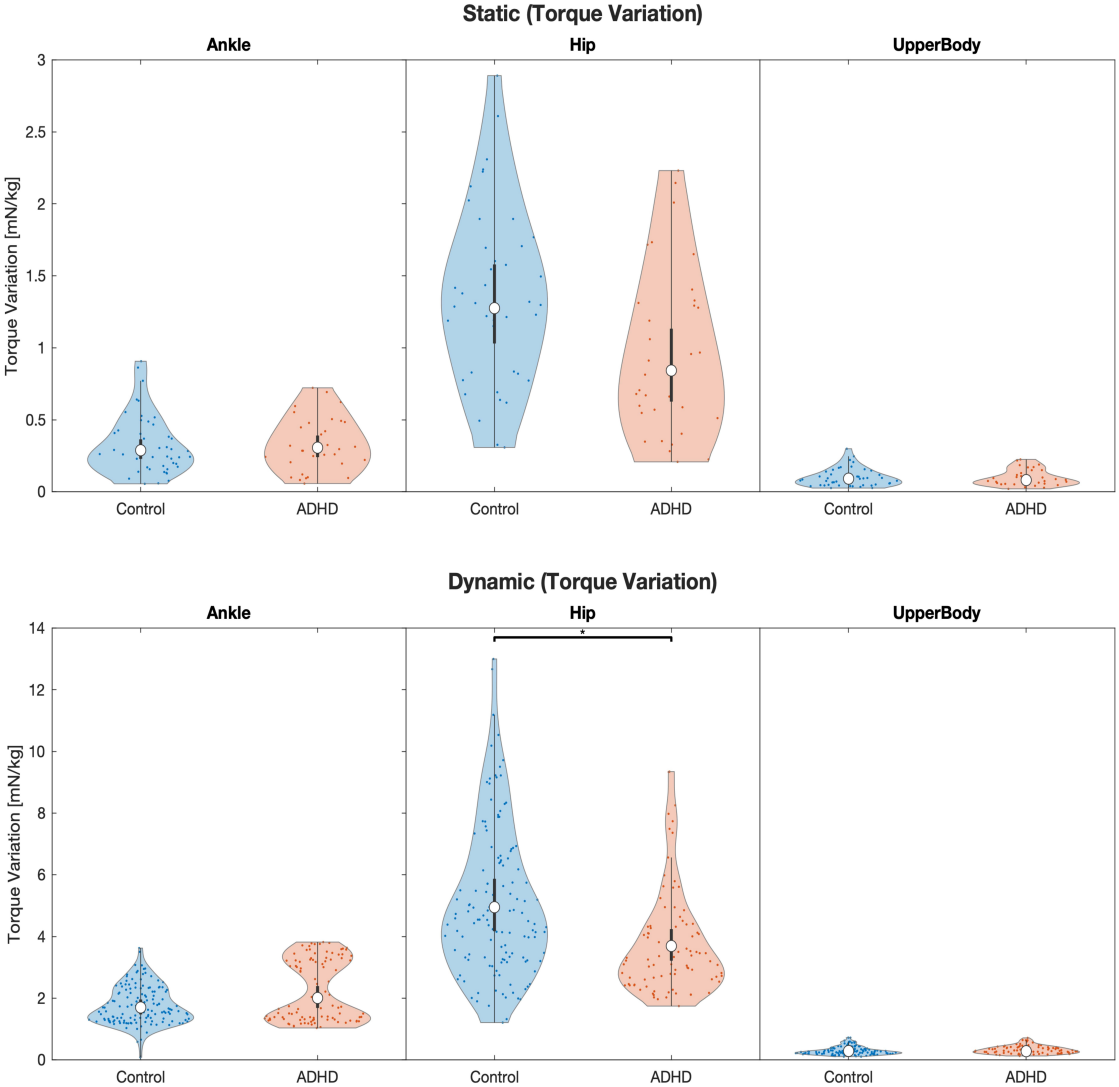
Violin plots of the linear model fit of joint torque variation for each joint group during static and dynamic balance tasks comparing children with and without ADHD. Empty circles indicate the estimated marginal mean (EMM), error bars denote the 95% confidence interval. Brackets indicate significant differences, asterisks denote the corresponding level of significance: ^*^p < 0.05, ^**^p < 0.01, ^***^p < 0.001. The units are in torque multiplied by 1,000 to obtain a more convenient number scale, giving Millinewton (mN) per meter body height and kilogram body mass, i.e., 1 mN^*^m/(kg^*^m) = 1 mN/kg.

Significant interaction effects on the *torque amplitude* in the one-leg stand (*F* = 10.01, *p* < 0.001, $\eta_{p}^{2}$ = 0.08), on the *torque variation* in the one-leg stand (*F* = 3.79, *p* = 0.02, $\eta_{p}^{2}$ = 0.03), and on the *torque variation* in the balance beam task (*F* = 5.39, *p* = 0.005, $\eta_{p}^{2}$ = 0.02), show that the two groups were affected differently in terms of torque generation in the various joint groups (Table 1; Figures 1, 2). A subsequent *post-hoc* test revealed that for the *torque amplitude*, only ankle and upper body joint torques but not hip joint torques differed significantly between groups, with higher torque amplitudes found for the ADHD group in all four instances. Specifically, group differences were found for the static balance task in the ankle joint (*F* = 20.7, *p* < 0.001, $\eta_{p}^{2}$ = 0.08) and the upper body joints (*F* = 5.49, *p* = 0.04, $\eta_{p}^{2}$ = 0.02), and for the dynamic balance task in the ankle joint (*F* = 5.2, *p* = 0.05, $\eta_{p}^{2}$ = 0.007) and the upper body joints (*F* = 6.39, *p* = 0.04, $\eta_{p}^{2}$ = 0.009; Table 2; Figure 1). Descriptive statistics are provided in Table 3.

**Table 2:** Results of the *post-hoc* analysis comparing the ADHD group with the neurotypical group for the three different joint groups with respect to torque amplitude and variation in each task as displayed in Table 1.

	**Joint group**	**p_corr_**	**%diff**	**F**	** $\eta_{\text{p}}^{2}$ **	**Effect size**
**One-leg stand torque amplitude**	Ankle	**< 0.001** ^***^	20.28	20.7	0.08	Medium to large
	Hip	0.05	−9.07	3.84	0.02	Small
	Upper Body	**0.04** ^*^	7.54	5.49	0.02	Small to medium
**One-leg stand torque variation**	Ankle	0.74	6.28	0.11	0.0005	Very small
	Hip	0.11	−33.97	4.49	0.02	Small
	Upper body	0.84	−10.86	0.65	0.003	Very small
**Balance beam torque amplitude**	Ankle	**0.05** ^*^	22.15	5.2	0.007	Small
	Hip	0.46	4.93	0.54	0.0008	Very small
	Upper body	**0.04** ^*^	11.28	6.39	0.009	Small
**Balance beam torque variation**	Ankle	0.33	18.1	1.94	0.003	Very small
	Hip	**0.03** ^*^	−25.41	6.74	0.01	Small
	Upper body	0.76	−2.25	0.1	0.0001	Very small

Statistically corrected p-values are indicated by pcorr. Relative changes are indicated in percent by %diff. Effect sizes are interpreted according to Cohen's rule of thumb. Asterisks denote the corresponding level of significance: ^*^p < 0.05, ^**^p < 0.01, ^***^p < 0.001. Significant results are in bold font and gray shade.

**Table 3:** Estimated marginal means (emMeans) with lower and upper limits of the 95% confidence interval comparing children with and without ADHD (neurotypical).

	**Group**	**Neurotypical**	**ADHD**
	**Joint Group**	emMean (95% CI)	emMean (95% CI)
**One-leg stand torque amplitude**	Ankle	1.54 (1.48, 1.6)	1.85 (1.73, 1.99)
	Hip	2.99 (2.79, 3.2)	2.72 (2.55, 2.89)
	Upper body	0.35 (0.33, 0.38)	0.38 (0.35, 0.41)
**One-leg stand torque variation**	Ankle	0.29 (0.23, 0.36)	0.31 (0.24, 0.39)
	Hip	1.28 (1.03, 1.58)	0.84 (0.63, 1.13)
	Upper body	0.09 (0.07, 0.12)	0.08 (0.06, 0.1)
**Balance beam torque amplitude**	Ankle	1.71 (1.5, 1.95)	2.09 (1.87, 2.34)
	Hip	3.86 (3.54, 4.21)	4.05 (3.7, 4.44)
	Upper body	0.56 (0.53, 0.58)	0.62 (0.58, 0.66)
**Balance beam torque variation**	Ankle	1.7 (1.47, 1.96)	2.01 (1.68, 2.39)
	Hip	4.95 (4.17, 5.87)	3.69 (3.21, 4.24)
	Upper body	0.29 (0.25, 0.33)	0.18 (0.25, 0.32)

Gray shaded columns show a significant difference.

## 4 Discussion

The aim of this study was to investigate the differences in postural strategies between children with and without ADHD during static and dynamic balance tasks. The results can at least partially confirm our hypothesis that there is a significant difference in torque amplitude and variation between children with ADHD and typically developing children in the tested ankle, hip, and upper body joint groups, during static and dynamic balance tasks. While significant differences between the two groups were found, these are not present across all joint groups and balance tasks. An investigation of general differences in task performance and balance ability between children with and without ADHD, preceded this analysis, yielding significant group differences in the task of dynamic balance (Christ et al., 2024).

For the assessment of static balance through a 25-s one-leg stand, group differences were found for torque amplitude, i.e., the amount of torque applied to the ankle and upper body joints differed significantly between the groups. While the groups differed by only 7.5%, with higher upper body joint torques produced by children with ADHD, the group differences were more pronounced for the ankle joint torque during the same task. The group of children with ADHD thereby showed a 20% higher joint torque amplitude than the neurotypical children. Correspondingly, the evaluation of our dynamic balance task in the form of an eight-meter balance walk over a narrow beam revealed significant group differences for the ankle and upper body joints in terms of torque amplitude. For this more challenging balance task, the group difference was accordingly marginally higher with a 22% higher ankle joint torque and 11% higher upper body joint torque amplitude in the ADHD group compared to the neurotypical children.

The ankle strategy is an activation pattern that effectively restores equilibrium by shifting the body's COM predominantly around the ankle joints (Horak and Nashner, 1986). Increased ankle torque generation during tasks of postural stability is associated with reduced neuromuscular control or inefficient movement patterns, such as corrective use of certain muscles to compensate for insufficient strength, flexibility, or coordination (Pozzi et al., 2015; Fok et al., 2021). Deficits in proprioception and neuromuscular control are common among children with ADHD (Hassan and Azzam, 2012; Shum and Pang, 2009; Izawa et al., 2012; Iglesias et al., 2014; Stray et al., 2015). A weakness in neuromuscular control among ADHD-affected children may manifest as reduced precision in muscle activation (Stray et al., 2015), which could in turn interfere with the adjustment of joint torques involved in maintaining balance and stability. Likewise, impaired proprioception or processing of sensory inputs within the patient group is likely owed to the compromised interaction among inputs from the vestibular, visual, and somatosensory systems, which feed the postural control system with orientation information and maintain body equilibrium (Guskiewicz and Perrin, 1996). However, it is also crucial to emphasize that high joint torques do not inevitably imply a shortcoming. In fact, most sports require high torque generation for efficient movement control and injury prevention.

Our findings of increased torque production in the upper body joints in the ADHD group suggest that affected children exhibit significantly more head, arm, and trunk movements during static and dynamic balance than typically developing children. Moving the upper body, especially the arms is a characteristic strategy to maintain or regain balance (Milosevic et al., 2011; Shafeie et al., 2012; Cheng et al., 2014; Patel et al., 2014; Boström et al., 2018; Objero et al., 2019). However, this strategy seems to be used to some extent by both, the neurotypical and ADHD-affected children as the group difference of 7.5% in the one-leg stand task is not as large as might be expected. A group difference of more than 11% on the balance beam task suggests that as task difficulty increases, the groups diverge further in their need for compensatory arm movements. Difficulties in long-term postural control have previously been observed in adult patients with ADHD, whose postural deficits were linked to symptoms of hyperactivity and impulsivity (Jansen et al., 2019). Similarly, attention regulation is often affected, so it is conceivable that the patient group may have difficulties in consciously controlling movements due to the nature of these symptoms, resulting in increased or inconsistent joint torques.

Within the 25-s single-leg standing task, no significant group differences were found for joint torque variation, our measure of the amount of change of torque for any joint group. This is somewhat surprising as the often uncoordinated and imprecise movements presented by children with ADHD would suggest that especially the measure of torque variation would reflect this. In contrast to that, it is the group of typically developing children that presents a 34% higher torque variation around the hip during the task of static balance. These findings are not in line with Mao et al. (2014) who noticed that children with ADHD, unlike their healthy controls, continuously changed their patterns of movement to find the correct balance strategy in a dynamic horseback riding task. Although not significant, this rather large group difference is interesting as it suggests distinctive tactics to restore equilibrium between the two groups. A similar observation to the static balance task was made in dynamic balancing, where the group difference was in fact significant with a 25% higher hip torque variation in the neurotypical group. Moreover, the 9% higher hip torque amplitude of the neurotypical group during static balance is noteworthy as this difference is trending toward significance.

The discovery of restricted hip movement is not completely novel and has already been made by Stray et al. (2009) where 80% of the ADHD patients displayed limited hip movement during a passive movement test, while this was not shown by the group of typically developing children. The authors conclude that this restricted hip movement may be a direct consequence of the balance problems linked to ADHD, as these are related to difficulties in keeping the torso in an upright position. They argue that the observed hip stiffness results from the extreme use of gross movement muscles like the erector spinae and psoas major muscles to compensate suboptimal proximal stabilization (Stray et al., 2009). Therefore, many ADHD patients seem to have induced biomechanical adaptations leading to strategies that involve more ankle torque to counterweigh the difficulty to create hip torque. This adaptation, however, is rather undesirable as the hip strategy seems to be more efficient than the ankle strategy for the restoration of balance. Regarding our findings, it is therefore thinkable that only the neurotypical subjects were able to generate sufficiently large compensatory hip torques as a way to regain stability. The hip strategy specifically involves activating the large muscles of the thighs and trunk to compensate for loss of balance (Horak and Nashner, 1986). Early activation of hip and trunk muscles during challenged translation on a narrow surface has previously been reported in healthy adults (Henry et al., 1998). Although hip and ankle strategy are two distinctive and similarly important methods, it is known that especially for more demanding balancing tasks and faster perturbations the hip strategy seems to be more efficient in stabilizing the body in terms of balance recovery and muscle stress compared to the ankle and upper body strategies (Runge et al., 1999).

Another interesting theory for hip stiffness and thus reduced torque generation in ADHD subjects is related to the brain's dopamine deficiency. Dopamine is an important neurotransmitter strongly involved in movement, motivation, and attention inter alia (Ungerstedt et al., 1982). Parkinson's disease, another condition in which low dopamine concentration in the brain plays an essential role, is characterized by symptoms like rigidity or stiffness (Heisters, 2011). Low brain dopamine levels may hence be another reasonable explanation for the observed hip stiffness in ADHD. Understanding why this stiffness manifests around the hip more than in other body regions requires further research.

Pertaining to brain regions involved in ADHD, not only disturbances in the prefrontal cortex and basal ganglia, but notably also impairments of the cerebellum are known to contribute to motor deficits such as balance or muscle tone regulation in the subject group (Goetz et al., 2014). Numerous neuroimaging studies report a reduction in cerebellar volume in ADHD patients compared to typically developing subjects (Castellanos et al., 1996; Krain and Castellanos, 2006; Mackie et al., 2007; Bledsoe et al., 2009). Since the cerebellum has a substantial function in postural control, sensory integration, and motor coordination, differences in balance strategies and thus differences in resulting torque generation between the groups of ADHD affected and neurotypical children during tasks of static and dynamic balance are plausible. Besides, excessive, unregulated movements may be owed to weakened impulse regulation in connection with cerebellar deficits (Jansen et al., 2019).

Moreover, a notable finding in our data is that age turned out to have a small but significant effect on torque amplitude in both balance tasks, while it did not significantly affect the torque variation. Many children with ADHD display a delay in motor development and compromised mechanisms of somatosensory integration (Rosa Neto et al., 2015). Yet, there seem to be age-related differences in torque generation among children aged 8 to 12 years.

The present study has several limitations. There is currently no standard data or uniform calculation method for the use of ankle, hip, or upper body strategies during balance tasks in children. Conclusions on increased or reduced joint torque amplitudes and variations can therefore only be drawn with respect to our relatively small control group of neurotypical children, of which we also lack standard data. Another limitation is that small faults in performance, such as losing balance and slipping of the balance beam, certainly create larger torques, which may be a confounding variable. However, such faults happened only rarely and were observed among both groups. A further limitation concerning the methodology is that the inverse dynamics simulation used to model the joint torques has to make specific assumptions to overcome the motor redundancies of muscular activation patterns. These assumptions, however, are potentially tailored to a neurotypical population and may or may not differ between neurotypical and non-neurotypical subjects. The small sample sizes and unequal gender distribution of the two groups are not necessarily representative of a broader ADHD population. Literature suggests that balance performance may differ among boys and girls (Nolan et al., 2005; Mickle et al., 2011). Although this evidence was shown for postural sway it is not unlikely that the same may apply to postural strategies in terms of joint torques, thus the findings must be interpreted with care. However, our results are useful to make deductions on boys and girls with and without ADHD on a broader age spectrum of 8 to 12 years. Another potential limitation is that the study did not differentiate between subtypes of the disorder, which could be improved by adding this element as a statistical covariate. However, postural stability in young ADHD patients is more dependent on the comorbid psychiatric disorders than on subtypes (Ghanizadeh, 2011). In summary, future studies would need to include a larger number of subjects and, if possible, collect detailed information on the demographic and clinical characteristics of the patient group to strengthen the applicability of the findings. A strength of our study is that all children in the ADHD group were medication-naïve, thereby excluding the possibility that postural stability is confounded by symptom-reducing medication.

## 5 Conclusion

Although during static and dynamic balance tasks various postural strategies are typically engaged simultaneously, our findings indicate that in a direct group comparison, children with ADHD tend to create torque predominantly around the ankle joints to stabilize their body, while they may not be able to rely on proximal stabilization from the hip, possibly due to high muscle tone and stiffness in this area. The larger torques observed when shifting the body COM around the ankle joints may result from reduced neuromuscular control, compromised integration of proprioceptive inputs, and inefficient movement patterns, commonly present in ADHD patients as reported by related literature. However, further research is warranted to confirm this connection. Likewise, increased movements of head, arms, and trunk during balancing may be related to symptoms like hyperactivity and impulsivity. These deficits in postural control are likely associated with a reduced cerebellar volume commonly reported in ADHD patients. While the amplitude of ankle and upper body torques varied notably between the two groups, no differences were observed for torque variation for these two joint groups. For the hip joint, lower torque variations were presented by the ADHD group compared to neurotypical group, implying very little hip movement during both balance tasks. Children with ADHD may have adopted postural strategies with a high involvement of ankle torque to compensate for their potential hip stiffness. Comparable to Parkinson's disease, hip rigidity in ADHD patients may be linked to dopamine deficiency in the brain, which, however, remains to be confirmed by future research.

Differences in joint torques during balance tasks between children with and without ADHD are the result of several factors, ranging from motor to sensory and even cognitive challenges. Creating joint torque to stabilize the body and control movements is beneficial and fundamental in many sports and daily activities. Children with ADHD may have difficulties efficiently employing and combining postural strategies to the requirements of a given situation. Therefore, assessing postural control within the affected group is an essential step in creating therapeutic interventions and training programs tailored to the precise needs of each individual so that postural stability is supported, whereby injury risk is minimized and wellbeing sustained.

## Data Availability

The datasets presented in this study can be found in online repositories. The names of the repository/repositories and accession number(s) can be found at: https://doi.org/10.17879/23908523699.
